# One Cell at a Time: Advances in Single-Cell Methods and Instrumentation for Discovery in Aquatic Microbiology

**DOI:** 10.3389/fmicb.2022.881018

**Published:** 2022-05-23

**Authors:** Vesna Grujcic, Gordon T. Taylor, Rachel A. Foster

**Affiliations:** ^1^Department of Ecology, Environment and Plant Sciences, Stockholm University, Stockholm, Sweden; ^2^School of Marine and Atmospheric Sciences, Stony Brook University, Stony Brook, NY, United States

**Keywords:** mass spectrometry imaging, Raman microspectroscopy, single-cell activity, single-cell genomics, single-cell genomics, single-cell transcriptomics, phenotypic plasticity, intra-population variability

## Abstract

Studying microbes from a single-cell perspective has become a major theme and interest within the field of aquatic microbiology. One emerging trend is the unfailing observation of heterogeneity in activity levels within microbial populations. Wherever researchers have looked, intra-population variability in biochemical composition, growth rates, and responses to varying environmental conditions has been evident and probably reflect coexisting genetically distinct strains of the same species. Such observations of heterogeneity require a shift away from bulk analytical approaches and development of new methods or adaptation of existing techniques, many of which were first pioneered in other, unrelated fields, e.g., material, physical, and biomedical sciences. Many co-opted approaches were initially optimized using model organisms. In a field with so few cultivable models, method development has been challenging but has also contributed tremendous insights, breakthroughs, and stimulated curiosity. In this perspective, we present a subset of methods that have been effectively applied to study aquatic microbes at the single-cell level. Opportunities and challenges for innovation are also discussed. We suggest future directions for aquatic microbiological research that will benefit from open access to sophisticated instruments and highly interdisciplinary collaborations.

## Introduction

Microbes play pivotal roles in marine and freshwater ecosystems. Microbes often live in very complex communities and engage in a myriad of interactions with each other (inter- and intra-species) and their environment. As minute nutrient processing factories, microbes are essential to life-sustaining biogeochemical cycles. Consequently, learning more about their physiology and functional role in the environment and the factors that influence population responses is vitally important.

Since many microbial (bacteria and archaea) populations primarily reproduce asexually, their gene pools should be represented theoretically by a large number of identical individuals. However, genomic changes due to horizontal gene transfer, mutations, or transposon insertions occur in individuals, frequently, and are not shared by an entire population’s cohort. Therefore, microbial populations sharing a core genome likely harbor substantial plasticity in their genetic, physiological, and behavioral traits. This phenotypic plasticity represents an adaptive opportunity for individuals to respond to changing or heterogeneous microspatial environments (Menden-Deuer and Rowlett, 2014). Meanwhile, responses of individual members are not necessarily normally distributed around a population average (Llamosi et al., 2016). Thus, mean trait values may not provide good accurate prediction performance. Therefore, single-cell level studies are essential to resolve phenotypic plasticity within populations, unbiased by signal averaging inherent to bulk measurements of heterogeneous populations.

**Figure 1:**
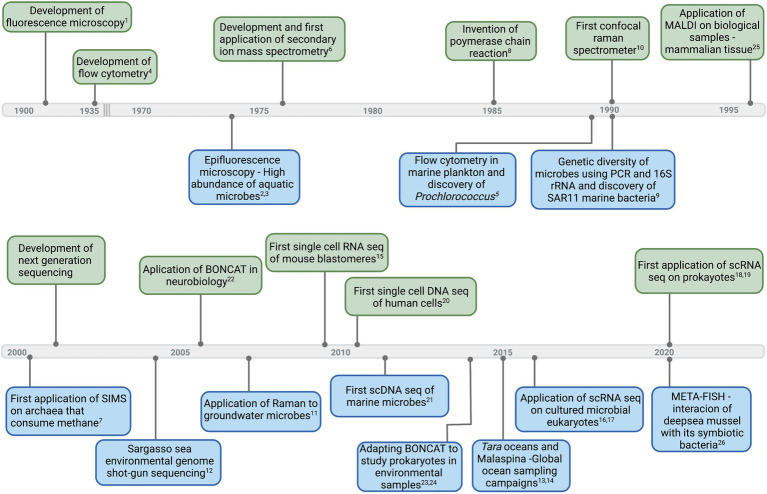
Timeline of select major developments in instruments and technologies (upper side of the timeline—light green) that led to first applications and important breakthroughs in aquatic microbial ecology (down side of the timeline—light blue). Corresponding references: ^1^(Renz, 2013), ^2^(Francisco et al., 1973), ^3^(Hobbie et al.,1977), ^4^(Moldavan, 1934), ^5^(Chisholm et al., 1988), ^6^(Clement et al., 1977), ^7^(Orphan et al., 2001), ^8^(Bartlett and Stirling, 2003), ^9^(Giovannoni et al., 1990), ^10^(Smith et al., 2016), ^11^(Huang et al., 2007), ^12^(Venter et al., 2004), ^13^(Pesant et al., 2015), ^14^(Duarte, 2015), ^15^(Tang et al., 2009), ^16^(Kolisko et al., 2014), ^17^(Liu et al., 2017), ^18^(Blattman et al., 2020), ^19^(Kuchina et al., 2021), ^20^(Navin et al., 2011), ^21^(Stepanauskas, 2012), ^22^(Dieterich et al., 2006), ^23,24^(Hatzenpichler et al., 2014; Samo et al., 2014), ^25^(Caprioli et al., 1997), and ^26^(Geier et al., 2020). Graphics created with BioRender.com.

Many advances in microbial ecology have depended upon adopting methods from far-ranging fields (Figure 1). Often methods are combined with fluorescent tags, isotopic labels, and high-resolution imaging. From single cells to assemblages, robust and highly resolved single-cell measurements have unveiled incredible functional, metabolic, and genetic diversity of aquatic microbes. In this perspective, we highlight several single-cell methodologies, with an emphasis on molecular imaging (revealing the native chemistry of the sample) and sequence-based applications. We present some challenges when adapting methods and instrumentation from other fields (Figure 1) and we offer suggestions for future directions in single-cell methodologies for aquatic microbes.

## Imaging and Measuring Microbial Activity and Interactions by Mass Spectrometry

By combining a variety of analytical imaging tools, aquatic microbiologists have interrogated and identified microbes and their interactions over the past several decades. For example, advances in Mass Spectrometry Imaging (MSI) have enabled remarkable discoveries (Figure 1). Herein, we highlight two MSI approaches: Matrix-Assisted Laser Desorption/ionization and secondary ion mas spectrometry (SIMS). For technical details, we refer readers to recent reviews (Watrous and Dorrestein, 2011; Dunham et al., 2017; Mayali, 2020; Zhu et al., 2021).

**Figure 2:**
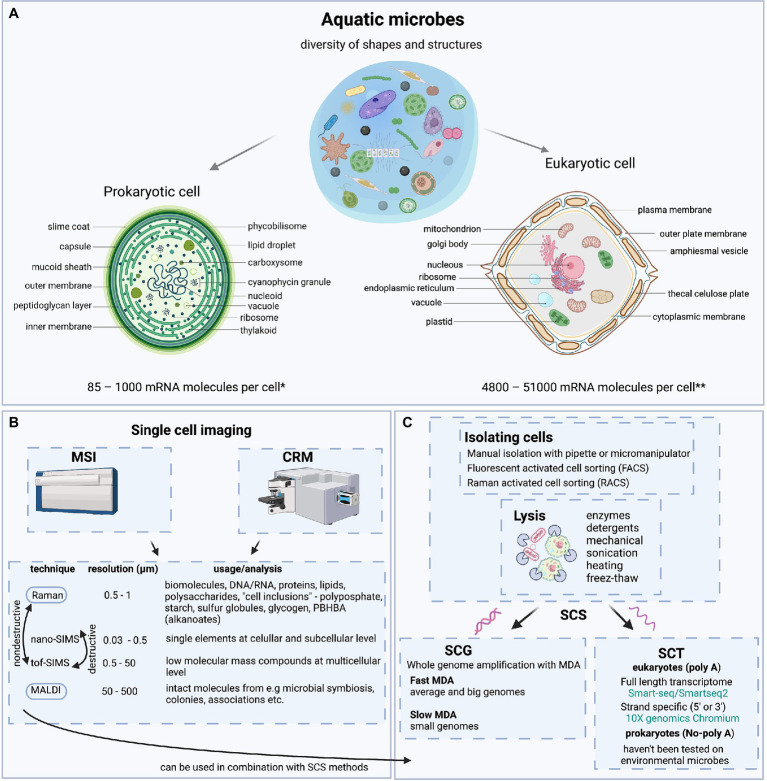
Overview of Aquatic Microbes, MSI and SCS workflows in Aquatic Microbiology. **(A)** Diversity of shapes and structure present in aquatic microbial cells. Prokaryotic cell architecture is illustrated using unicellular cyanobacteria as an example. Eukaryotic cell architecture is illustrated using a thecate non-flagellated dinoflagellate example. Amounts of mRNA in prokaryotes and eukaryotes are based on estimates. * Based on RNA recovery from natural bacterioplankton communities of coastal waters (Moran et al., 2013 and reference therein). **Based on RNA recovery from culture of haptophyte (*Prymnesium parvum*) and dinoflagellate (*Karlodinium veneficum*) (Liu et al., 2017). **(B)** Single cell imaging techniques; comparing capabilities of mass spectrometry imaging (MSI) and confocal Raman microspectroscopy (CRM). Techniques are arranged according to spatial resolution and what molecular/cellular structures they can analyze. Raman and MALDI are non-destructive techniques so they could be used in combination with SCS methods. **(C)** Diversity of shape, size, and structure of microbial cells presents challenges to separation and isolation of cells. Most common ways of isolating cells are presented. Lysis is another problem arising from diverse and thick cell walls. The most common ways to lyse cells are presented. Single cell sequencing techniques, including single cell genomics (SCG), single cell transcriptomics (SCT) and an assortment of protocols and technologies used in SCS methodologies are presented. Most common method for SCG gene amplification in aquatic microbial ecology is with MDA reaction. Depending on genome size and difficulties in cell lysis, different MDA amplification techniques can be applied and optimized (Ciobanu et al., 2022). SCT technologies tested on cultured aquatic microbial eukaryotes include those for full length transcriptome generation (Kolisko et al., 2014; Liu et al., 2017) and those that generate only one side of transcriptome (3’ or 5’ strand specific) (Ma et al., 2021). SCT has not yet been tested on aquatic prokaryotes although available technologies have been tested on other bacterial representatives. Graphics and illustrations made with BioRender.com.

Mass Spectrometry (MS) has continually advanced since its invention (Langmuir, 1945). In the past two decades, several applications of MSI have developed, impacting many fields, including aquatic microbiology (Watrous and Dorrestein, 2011). In MSI, ions (or molecules) are mapped from sample surface coordinates (x-y position) at nano to microscales (nm to μm) that are relevant to microbial consortia. Signal intensity, displayed in pseudo-color, is overlain on x-y coordinates that provide positional information (Maloof et al., 2020). MS instruments can operate either under high vacuum or atmospheric pressure (AP). MS methodologies also differ in levels of sample destruction, mass resolution, spatial resolution, and compatibility with isotopic labels (Figure 2B; Supplementary Box 1).

Two decades ago, MSI emerged as a groundbreaking high-resolution tool for the materials and biological sciences and was widely applied in peptide and proteomics studies in biomedical, immunology, and cancer research. In its first biological demonstration, MSI enabled two-dimensional mapping on a mammalian tissue section using MALDI time of flight (ToF) MS (Caprioli et al., 1997). A recent similar example in aquatic microbiology developed META-FISH or the combination of AP-MALDI with fluorescent *in situ* hybridization (FISH) (Geier et al., 2020). Using this technique, interactions between deep-sea mussels and intracellular symbiotic consortia (sulfur and methane oxidizers) could be differentiated and linked spatially to individual partners (Geier et al., 2020).

Both MALDI-MSI and SIMS have a distinct advantage over traditional bulk measurements in that analytes are measured on intact chemically fixed or fresh-frozen preparations (e.g., tissues and cells) and not from extracted materials. However, unlike SIMS, MALDI-MSI does not require labels (e.g., stable isotopes or radiotracers) and analytes are identified based on mass-to-charge (m/z) ratios of entire molecules or fragments thereof. Numerous applications of MSI in biomedicine, immunology, and food sciences have studied inter-domain metabolic interactions, e.g., between pathogenic bacteria and fungi (Moree et al., 2012), metabolic profiling and quorum sensing in bacterial biofilms (Ravindran et al., 2018; Santos et al., 2018), and distribution and transfer of drugs and micropollutants in biological samples (Thomen et al., 2020). Aquatic microbes often live in exceedingly complex consortia (e.g., aggregates, metazoan guts, biofilms, and symbioses) and engage in inter- and intra-species metabolite exchange (Azam and Malfatti, 2007). Thus, MALDI-MSI can be applied to study similar interactions, and since it is a label-free approach, metabolites are directly mapped in cells of interest. Although lateral spatial resolution (3 μm) limits its application to many aquatic microbes, MALDI-MSI could be applied to holobionts or archived samples (e.g., sediment traps and plankton tows) to improve our understanding of coordinated responses of microbial communities to natural and environmental perturbations (e.g., climate change and oil spills).

SIMS is another widely used analytical technique originating from material sciences. This first introduction to aquatic microbiology combined FISH with measures of ^13^C depletion made on a 1,280 large-geometry (LG-) SIMS instrument, which has low lateral resolution, and as such only aggregates were resolvable (Orphan et al., 2001). Shortly thereafter three independent first stable isotope probing (SIP) studies coupled the commercially available nanoSIMS 50/50 L platform with FISH (Behrens et al., 2008; Li et al., 2008; Musat et al., 2008), each utilizing probes with different halogen reporters with a specific detectable mass. Each study demonstrated the capacity to simultaneously link labeled substrate assimilation with the individual microbial phylotypes. In the past decade, a number of major discoveries used nanoSIMS alone, FISH-SIMS, or complementing SIMS with other single-cell physiological measures (e.g., Bioorthogonal Non-canonical Amino Acid Tagging (BONCAT) for protein translation) (Hatzenpichler et al., 2014; Samo et al., 2014; Pasulka et al., 2018) (Figure 1). In fact, nowadays, nanoSIMS previously used as a stand-alone method is more often a component of a larger study.

An alternative method to FISH-SIMS is called CHIP-SIP, which similarly allows simultaneous measurements of multiple isotope labels, but uses a high-density microarray (e.g., 2,500 oligonucleotides/0.75 mm^2^) or a chip. Thus, isotopic enrichment is visualized on a microarray spot, which is specific for a target microbial phylotype. So far it has been applied to rRNA targets. Targeting functional genes should be feasible for CHIP-SIP, but target genes need to be highly expressed for microarray detection. Moreover, throughput could be heightened using a SIMS1280 platform which is more stable and can operate in an automated mode. Both nanoSIMS and SIMS1280 are capable of storing coordinates on the sample stage, allowing the user to return to the same spot; however, re-calibration of the masses can be required.

Herein, we briefly highlight a few challenges to consider, and solutions when known, in applying MALDI and SIMS to aquatic microbiology. Although SIMS techniques achieve horizontal spatial resolutions within 10s to 100 s of nm, they mainly provide isotopic signatures, elemental concentrations, and fragment ions, whereas MALDI-MSI can identify intact biomolecules such as sugars, amino acids, lipids, glycans, and even peptides and proteins. MALDI-MSI is capable of spatial resolutions of 1 to 10s of µm.

MSI analyses are routinely applied to flat surfaces. Sample surface topography can be challenging for these ablative technologies because the ion beam has a narrow focal plane (nm to μm). Thus, two-dimensional MSI mapping of complex microbial forms are potentially problematic when portions of cells are above or below the beam’s focal plane. Hence, some investigators embed cells and section with a microtome or grow cells on agar prior to analyses; note agar is only compatible with MALDI.

As in any analytical approach, efficient protocols for sample preparation are crucial for success. MALDI has more relaxed sample preparation requirements, is highly automated, and includes workflows and software for programing raster grids (Watrous and Dorrestein, 2011). However, some of the challenges still lie within identification of the metabolites/proteins and elaborate sample preparation techniques (e.g., for high-resolution measurements, protein digests, or even correlative imaging approaches).

SIMS imaging employs a hard or destructive ionization strategy. SIMS sample preparation largely depends on sample type and analytical application. For example, isotope dilution caused by chemical fixation and FISH protocols is a concern specific to FISH-SIMS (Musat et al., 2014). Cryopreservation can replace chemical fixation; however, it requires specialized instruments. Critical to SIMS analyses is the ability to mark regions of interest in a field of view, this is particularly difficult with mixed populations. Additionally, too much handling can disrupt cell aggregates and/or modify community architecture (e.g., filtering cells on top of each other). Estimating single-cell uptake rates is common with SIMS data and requires knowledge of the initial elemental content. Recently, Stryhanyuk et al. (2018) provided a model-based solution to correct for isotope dilution and alteration during sample preparation, including expected isotope fractionation patterns and alternatives for estimating cellular biovolume.

## Investigating Microbial Composition and Activity Using Confocal Raman Microspectroscopy (CRM)

Like infrared spectroscopy, Raman is a form of vibrational spectroscopy but relies on on inelastic scattering of monochromatic light to provide specific chemical information (Figure 2B). Unlike IR spectroscopy and MSI platforms, CRM can analyze aqueous samples and is insensitive to most ions dissolved in seawater. Sample preparation requirements for Raman spectroscopy are relaxed, i.e., dried, live, preserved, and stained, and frozen samples can all be interrogated under standard laboratory environmental conditions. CRM is also capable of yielding label-free chemical information on aquatic microbiota. Samples irradiated with laser light primarily re-emit Rayleigh scattered photons, but a small portion of laser photons (10^−6^–10^−7^) transfer energy to specific molecular sites, scattering photons at different frequencies (Raman scattering). Energy differences between excitation photons and Raman-scattered photons directly relate to specific chemical bonds. The working platform of most Raman microspectrophotometers is a modified epifluorescence microscope coupled to one or more lasers and a Raman spectrograph which images Raman-scattered photons into a CCD detector (see Taylor, 2019; Lee et al., 2021). Spectra of complex materials yield multiple peaks whose positions are diagnostic of specific molecular bonds and heights/areas are proportional to analyte concentration.

Unique to CRM, a researcher can identify specific microbial taxa within a microscopic field using FISH and the instrument’s epifluorescence optics and then immediately switch optical paths to obtain Raman spectra from each target in a matter of seconds to minutes (Raman-FISH) (Huang et al., 2007). The automated microscope stage can visit each target using stored x-y coordinates or create fixed-point gridded maps (Taylor, 2019; Weber et al., 2021), similar to grid systems in MSI. Sampling by Raman spectroscopy is usually non-destructive, so samples can be subjected to other downstream analyses. For example, researchers recently developed an automated optofluidic platform for Raman-activated cell sorting (RACS) that rapidly analyzes bacterial cells in a fluid stream and segregates metabolically active cells based on Raman signature for deuterium assimilation (Berry et al., 2015; Hatzenpichler et al., 2020; Lee et al., 2021). Sorted cells can then be subjected to phylogenetic identification or single-cell genomics.

In contrast to SIMS, and similar to MALDI, CRM provides information on molecular species (“molecular fingerprints”), rather than elemental analyses. CRM closes a critical gap between SIMS and MALDI by providing higher molecular specificity than SIMS and higher spatial resolution than MADLI and even enables live cell imaging which MSI approaches lack (Ryabchykov et al., 2018). When applied to biological materials, CRM primarily yields information on polymeric materials, such as proteins, nucleic acids, lipids, polysaccharides, starch, and polyphosphates (Figure 2B). Importantly, CRM can detect isotopic substitutions in analytes, thereby enabling SIP-Raman experiments to track movement of major elements from dissolved pools into specific microbial cellular pools on a single-cell basis. For example, Li et al. (2012) demonstrated that resonance Raman spectra of carotenoids can quantify dissolved inorganic carbon assimilation by individual cyanobacteria and coastal phytoplankton cells incubated with ^13^C-bicarbonate. Building on this advance, Taylor et al. (2017) and Weber et al. (2021) demonstrated that SIP-Raman accurately measures growth rates in individual cells of photoautotrophs and heterotrophs, respectively. Growth rates have also been reported in nanoSIMS studies based on enrichment ratios (Foster et al., 2011, 2021). SIP-Raman has also been applied to food web studies, tracing movement of carbon from prey to predator (Li et al., 2013). Taking SIP-Raman a step further, Yakubovskaya et al. (2021) demonstrated that individual viruses inherited the ^13^C-isotopic signatures of their labeled host, *Emiliania huxleyi*. Similarly, a viral-BONCAT method was used in concert with nanoSIP to track N and C transfer from host to virus during lysis (Pasulka et al., 2018). These technological advances enable more detailed examination of trophic interactions, viral infection, symbioses, and to directly assess intra- and inter-population variability in growth phenotypes and trait plasticity in microbial communities.

All analytical techniques have their inherent strengths, limitations, and challenges. CRM is no exception. The ability to extract detailed chemical information from individual live or fully hydrated microbial cells under standard laboratory conditions is one of the most outstanding strengths of CRM. This attribute enables mapping distributions of molecular pools within undistorted and unaltered cells in two and three dimensions as well as to track movement of stable isotope tracers through time (e.g., Wakisaka et al., 2016; Taylor, 2019; Weber et al., 2021). Raman scattering, however, is an inherently weak phenomenon (1 in 10^6^ to 10^7^ photons scattered) meaning that many analytes will be below the method’s detection limits. Nonetheless, several signal enhancement technologies are currently under development, recent summaries can be found in Lee et al. (2021). Spatial resolution is diffraction-limited to about 350 nm at best with standard CRM (Lee et al., 2021). Technologies to improve spatial resolution of CRM are on the horizon and also described elsewhere (e.g., Taylor, 2019). Biological materials tend to fluoresce which can be a significant challenge to applying CRM to aquatic microbes. When present, the fluorescent cross-section greatly exceeds that of Raman scattering and consequently masks Raman signals. Several approaches are available to overcome the fluorescence challenge and are described in Huang et al. (2007), Taylor (2019), Yakubovskaya et al. (2019), and Lee et al. (2021).

## Investigating Application of Single-Cell Sequencing (SCS) To Aquatic Microbial Ecology

SCS was selected by Nature as “Method of the Year” in 2013 (Nature Methods, 2014) and later in 2020 as a technology to watch; likewise MALDI-MSI was among the seven technologies acknowledged in 2021 (Landhuis, 2021). Since then, SCS has made great successes and broad applications in biomedicine (Yasen et al., 2020). SCS refers to either single-cell DNA (single-cell genomics, SCG) or RNA (single-cell transcriptomics SCT) sequencing (Figure 2C). Numerous review articles describe current SCS protocols and challenges (Chen et al., 2017; Woyke et al., 2017; Ku and Sebé-Pedrós, 2019; Kaster and Sobol, 2020; Brennan and Rosenthal, 2021). SCS’s most important advantages, compared to bulk approaches (i.e., metagenomics and metatranscriptomics), are generating genomes and transcriptomes from low abundance species, determining metabolic potential of individual cells and linking them to their community (Cheng et al., 2019). Here, we review important considerations for SCS applications to environmental prokaryotes and microbial eukaryotes (protists) and highlight relevant examples. We recognize four major challenges: 1) isolation of individual cells, 2) uniform cell lysis across complex communities, 3) limitations in available technologies, and 4) low recovery and instability of nucleic acids.

SCG was used to generate one of the first draft thaumarchaeal genomes and revealed new features (e.g., chemotaxis and motility) which were previously unknown in these archaea (Blainey et al., 2011). SCG has also led to two new proposed prokaryotic superphyla (Rinke et al., 2013), revealed viruses that are consumed by marine protists (Brown et al., 2020) and resolved phylogenomics of aplastidic picozoa (Schön et al., 2021). Recently, Pachiadaki et al., (2019) applied SCG to exhaustively and non-selectively sequence aquatic prokaryotic genomes residing in just 0.4 ml seawater samples and reported on genomic complexity and organization of environmental microbial assemblages. However, SCG has significant limitations that challenge its application to a range of environmental microbes (Figure 2A).

Aquatic microbes live in complex environments where separation of cells poses a critical challenge to lysing a single cell. The most common ways to separate aquatic microbes are by dilution, single-cell pipetting, or flow cytometry coupled to fluorescent activated cell sorting (FACS) (Rinke et al., 2014), which tend to be labor intensive and can compromise genome quality. Recent advances in microfluidic cell separation represent a great high-throughput alternative (Murphy et al., 2017) and a detailed review of its application to plankton research is presented in Girault et al. (2019).

Variable cell lysis efficiency results in part from the very diverse nature of microbial cell walls; extracellular structures of aquatic prokaryotes and protists pose a significant challenge (Islam et al., 2017). For example, Gram-negative bacteria have an exterior plasma membrane made of lipopolysaccharides and phospholipids (Figure 2A). Cell walls of protists have diverse compositions: cellulose or calcareous plates, silica frustules, chitin, mineral cell walls, or unknown biochemical compositions (Corliss, 2002) (Figure 2A). Thus, developing a universal method to permeabilize cells is difficult, especially when working with uncultivable microbes. So far, lysis has been largely optimized for each microbe of interest separately. Moreover, lysis methods are usually adopted from mammalian cell protocols, which are inherently easier to lyse compared to aquatic microbes (Islam et al., 2017). Examples of successful lysis methods are presented in Figure 2.

Reproducible application of the multiple displacement amplification (MDA) procedure commonly used to amplify templates is challenging owing to introduction of chimeras and to incomplete or uneven coverage of the cells’ entire genome. Estimated genome completeness of single amplified genomes (SAGs) varies tremendously, e.g., 5–100% for prokaryotes (Rinke et al., 2013; Clingenpeel et al., 2014) and 9–55% for protists (López-Escardó et al., 2017). The thermostable mutant phi29 polymerase in the MDA step has been shown to improve amplification performance (Stepanauskas et al., 2017). Additionally, interpretation of SCG results can be facilitated by complementing with available metagenomics data (Mende et al., 2016; Arikawa et al., 2021). Hosokawa et al. (2017) demonstrated that microfluidics-based cell separation and MDA improved both completeness and decreased contamination rate for SAGs of soil bacteria. Recently, Ciobanu et al. (2022) published a successful and extensive protocol for SAG amplification of microbial eukaryotes.

SCT has been applied to environmental microbes far less often, and unlike SCG, it was initially applied to protists (Kolisko et al., 2014; Liu et al., 2017), because SCT methodologies were initially developed for eukaryotic cells. Consequently, the method relies on the 3′ poly-adenosine (poly-A) tail present in eukaryotic mRNA (Brennan and Rosenthal, 2021). However, using poly-A-based sequencing prevents studying microbial interactions between protists and prokaryotes (e.g., many planktonic symbioses and particle-associated microbiota). Few successful applications have been reported for aquatic microbes and major challenges remain, primarily how to adapt methods that were developed for mammalian cells.

Similar to SCG, reliable cell separation and lysis are challenging for the same reasons described above. Additional key considerations relate to RNA stability and mRNA concentrations. mRNA half-lives of prokaryotic and protists cells are much shorter than that of mammalian cells (Figure 2A). Half-life measurements of mRNA from the marine cultured bacteria are estimated to be from 2.4 to 10 min (Steglich et al., 2010; Steiner et al., 2019). Marine bacteria are estimated to contain 85–1,000 mRNA per cell and average of ~250 copies of mRNA per cell at any given moment (Moran et al., 2013). Data on mRNA concentrations and half-lives in protists are very limited and likely much more variable. In general, protist cells are presumed to contain more mRNA given their larger cell sizes. One study reported that the dinoflagellate *Karlodium veneficum* (cell length ~ 15 μm) and the haptophyte *Prymnesium parvum* (cell length ~ 8 μm) contained on average ~ 51,000 and ~ 4,800 mRNA molecules cell^−1^, respectively (Liu et al., 2017). These estimates are considerably lower than that reported in mammalian cells (*Homo sapiens*; 50,000–300,000 mRNA molecules cell^−1^) (Marinov et al., 2014). Additional problems with SCT are finding suitable statistical treatments and interpreting results due to the fact that microbes have fast cell cycles. Therefore, variation in individual gene expression can be substantial, impacted by many factors and hard to interpret.

SCT application to aquatic microbiology is still in its infancy. Thus far, protocol testing and method development have been on cultured eukaryotic microbial plankton for which reference genomes are available or on easily cultivated model organisms (*Chlamydomonas reinhardtii)* (Kolisko et al., 2014; Liu et al., 2017) (Ma et al., 2021). Recently, SCT was combined with bioinformatic tools to study genome architecture and protein evolution in uncultivable ciliates (Yan et al., 2019). Using SCT, a novel group of primitive dinoflagellates were discovered by manual isolation from a 600 m sample which showed a functional but reduced plastid (Cooney et al., 2020).

## Conclusion

Development and application of single-cell methods have greatly advanced in the last decade. We presented a glimpse into such advances by highlighting a small subset of single-cell technologies that have promising applications in aquatic microbial ecology. SCS methods combined with single-cell imaging (MSI and CRM) can provide unprecedented insights into the molecular composition of aquatic microbes and their microscale environment. While many of these methods were originally developed for other fields, they provide inspirational models for application to aquatic microbiology. Moreover, they are highly informative when applied in concert with other complementary methods (e.g., BONCAT and FISH). Many successful applications of MSI and SCS have relied on cultivable model systems, which remains challenging for environmental microbiological studies. Thus, increased efforts to isolate and cultivate target organisms, reliance on *a priori* sequence-based knowledge, improving and expanding identifications of metabolite and protein databases, and a fair amount of trial and error will be required to fully exploit the potential of the single-cell methods presented here.

Future aquatic microbiological research should continue to co-opt methods and instrumentation from other disciplines. One could integrate these highly precise single-cell measurements into cellular and biogeochemical models. Still to come is implementation of inter-calibration exercises between facilities and instruments and improving data repositories for MSI and CRM datasets that are similarly required as that for sequencing. Advances in single-cell applications will be facilitated by open access to data, instruments, and interdisciplinary collaborations, including those from distant fields.

## Data Availability Statement

The original contributions presented in the study are included in the article/Supplementary Material, further inquiries can be directed to the corresponding author.

## Author Contributions

VG and RF conceptualized and proposed the idea of the perspective. RF and GT provided background knowledge of mass spectrometry imaging and Raman Microspectroscopy. VG wrote the first draft of the manuscript. All authors contributed to the article and approved the submitted version.

## Funding

Contribution by RF and VG is supported by Knut and Alice Wallenberg Foundation grant to RF. GT acknowledges support from NSF grants OCE-1336724, OCE-1851368, and OCE-1924424 and a Gordon and Betty Moore Foundation grant #5064.

## Conflict of Interest

The authors declare that the research was conducted in the absence of any commercial or financial relationships that could be construed as a potential conflict of interest.

## Publisher’s Note

All claims expressed in this article are solely those of the authors and do not necessarily represent those of their affiliated organizations, or those of the publisher, the editors and the reviewers. Any product that may be evaluated in this article, or claim that may be made by its manufacturer, is not guaranteed or endorsed by the publisher.
