# Supplementary material for: One Cell at a Time: Advances in Single-Cell Methods and Instrumentation for Discovery in Aquatic Microbiology
Source: Front Microbiol. 2022 May 23;13:881018. doi: 10.3389/fmicb.2022.881018 (PMC9169044; doi:10.3389/fmicb.2022.881018)
Supplement: Supplementary file 1 [file Table_1.DOCX]

Supplementary Box 1. MSI is an analytical technique that generates gas phase ions from a sample (organic or inorganic) by the bombardment of ionization energy in order to determine their mass-to-charge ratio (m/z). Thus, a simplified version of a mass spectrometer consists of an ion source, ion optics, and a mass analyzer. MSI instruments are distinguished by how the ions are generated from the sample surface. Secondary Ion Mass Spectrometry (SIMS) uses a continuous beam of highly focused primary ions to bombard (or “sputter”) the sample surface. The sputtering of the primary beam emits secondary ions (atoms and clusters of atoms) that accelerate into the MS for analyses. SIMS uses hard ionization sources and is destructive and fragment molecules, and as such limited for smaller mass analyses. Matrix-assisted laser desorption/ionization MALDI-MS apply ions to the sample by a laser and is a soft ionization technique optimal for larger molecules. A distinct advantage of both approaches is the simultaneous detection of multiple masses, and when paired with imaging (e.g., MSI), the ability to map back the ions to a specific location on the sample surface. Notably, both approaches also allow label-free unbiased imaging of a sample as they often occur in non-model systems from aquatic/marine environments. SIMS operates at high vacuum (10^-5^ to 10^-10^ Torr), while MALDI can be run at atmospheric pressure (AP). NanoSIMS and SIMS1280 microprobe instruments require dry samples and have highest spatial resolution available (nm to μms); MALDI has a high sample tolerance and capable of measuring microbial colonies and tissue samples on solid media and has spatial resolution of 1000 μm.
